# Metabonomic analysis of ovarian tumour cyst fluid by proton nuclear magnetic resonance spectroscopy

**DOI:** 10.18632/oncotarget.6891

**Published:** 2016-01-12

**Authors:** Michael Kyriakides, Nona Rama, Jasmin Sidhu, Hani Gabra, Hector C. Keun, Mona El-Bahrawy

**Affiliations:** ^1^ Department of Surgery and Cancer, Imperial College London, London, United Kingdom; ^2^ Department of Histopathology, Hammersmith Hospital, Imperial College London, London, United Kingdom; ^3^ Department of Pathology, Faculty of Medicine, University of Alexandria, Alexandria, Egypt

**Keywords:** cyst, metabonomics, ovarian, tumour, metabolite

## Abstract

The majority of ovarian tumours are of the epithelial type, which can be sub classified as benign, borderline or malignant. Epithelial tumours usually have cystic spaces filled with cyst fluid, the metabolic profile of which reflects the metabolic activity of the tumour cells, due to their close proximity. The approach of metabonomics using ^1^H-NMR spectroscopy was employed to characterize the metabolic profiles of ovarian cyst fluid samples (*n* = 23) from benign, borderline and malignant ovarian tumours in order to shed more light into ovarian tumour and cancer development. The analysis revealed that citrate was elevated in benign versus malignant tumours, while the amino acid lysine was elevated in malignant versus non-malignant tumours, both at a 5% significance level. Choline and lactate also had progressively increasing levels from benign to borderline to malignant samples. Finally, hypoxanthine was detected exclusively in a sub-cohort of the malignant tumours. This metabonomic study demonstrates that ovarian cyst fluid samples have potential to be used to distinguish between the different types of ovarian epithelial tumours. Furthermore, the respective metabolic profiles contain mechanistic information which could help identify biomarkers and therapeutic targets for ovarian tumours.

## INTRODUCTION

Ovarian cancer is the second most common gynaecological malignancy worldwide and the fourth most deadly type of cancer in the UK. [1] Around 90% of ovarian tumours are of the epithelial type and the four most common histological subtypes are: serous, endometrioid, mucinous and clear cell carcinomas. [2] Epithelial ovarian tumours in general can be categorized as benign, borderline or malignant, depending on architecture, cytological atypia and the presence of stromal invasion. [3] The majority of borderline ovarian tumours behave in a benign fashion, but a small proportion may recur or show progressive disease in a manner similar to malignant tumours. [4] All types of epithelial ovarian tumours may typically include a cystic component which encapsulates variable amounts of cyst fluid. [5]

Even though during the past few decades there have been advances in surgery and taxol/platinum-based chemotherapies, the drop in mortality rates of patients suffering from ovarian cancer has been very modest. [6] This disappointing rate of progress can be partly attributed to the high relapse rate of patients with a drug-resistant disease [7], and the five year survival rate of patients with an advanced stage of the disease is only 5-30%. [8] Another reason why there has been a limited success in ovarian cancer therapy is the difficulty in detection at an early stage due to the lack of an adequate screening method as well as the late development of the symptoms. As a result ovarian cancer is often called the “silent killer”. [3, 9]

The ability to detect the presence of cancer at an early stage or to distinguish between the different types of tumours has been strengthened by the use of tumour biomarkers. Biomarkers can be detected in a variety of sample matrices but in the context of ovarian cancer; urine, serum or ovarian cyst fluids have been used in the past. [3, 10, 11]

Several attempts of identifying biomarkers for epithelial ovarian cancer have led to the discovery of several candidates but unfortunately none of the currently proposed biomarkers is of ideal sensitivity and specificity. Enzyme-linked immunosorbent assay (*ELISA*) of ovarian cyst fluids have indicated that the cytokines interleukin-6 (IL-6) and IL-10 exist at higher levels in patients with a more advanced stage of disease. [12] Another example of a potential biomarker is the glutathione S-transferase P1-1 enzyme which has been discovered to exist in higher levels in the ovarian cyst fluid of malignant tumours when compared to benign tumours. [13]

A relatively novel approach of detecting biomarkers, known as metabonomics, has been a major development, especially in the field of toxicology or with diseases such as cancer. Metabonomics was first described as: “the quantitative measurement of the dynamic multiparametric metabolic response of living systems to pathophysiological stimuli or genetic modification”. [14] Metabonomics thus studies the metabolic profiles of specific biological states or the metabolic response to a system change. It often relies on multivariate statistical methods to process the metabolic profiles, as well as identifying any significant patterns or molecular fingerprints which could serve as potential biomarkers. Nuclear magnetic resonance (NMR) spectroscopy is an often used method for obtaining metabolic profiles. [15] As a tool for metabolic profiling ^1^H-NMR spectroscopy has the advantages of requiring minimal sample preparation and possesses high reproducibility and quantitative precision. [16] The coverage of the metabolome is relatively low however due to lack of spectral resolution and inherently poor sensitivity. Mass-spectrometry based approaches, including liquid chromatography mass spectrometry, gas chromatography mass spectrometry and capillary electrophoresis mass spectrometry (LC-MS, GC-MS and CE-MS respectively), have greater sensitivity and are usually coupled to derivatisation/chromatography, allowing for greater coverage, but are therefore also subject to more interferences, matrix effects and sources of irreproducibility.

Tumour cells possess altered metabolic behaviour, such as a growing dependence on glycolysis and increased lactate production (the Warburg effect) [17], and increased rates of glucose uptake are linked to tumour aggressiveness. [18] Metabonomic analysis of serum, urine and ovarian cyst fluid samples from patients with epithelial ovarian tumours has been described in the literature and how it can provide information on the tumour-related alterations in metabolism in these patients. [10, 19] Because of the close proximity of the cyst fluid to the tumour micro-environment, ovarian cyst fluid samples could be the most informative matrix for a more complete metabolic profile of these tumours. ^1^H-NMR spectroscopic analysis on ovarian cyst fluid has led to the identification of several metabolites which seem to exist in higher levels in malignant tumour samples such as lactate, isoleucine, valine, 3-hydroxybutyric acid, methionine and alanine. [19]

In this study we investigate the metabolic profile of cyst fluid from benign, borderline and malignant ovarian epithelial tumours and show the potential of high throughput analysis of ovarian tumour cyst fluid in identification of biomarkers that discriminate between the different tumour categories.

## RESULTS

Cyst fluid samples from 23 ovarian epithelial tumours were studied, including 8 benign, 5 borderline and 10 malignant tumours were analysed. Histopathological details of the studied tumours are presented in Table 1.

**Table 1:** Overview of patients’ tumour type, grade and FIGO stage

Patient	Tumour type	FIGO stage	Grade
1	Benign mucinous cystadenoma		
2	Benign mucinous cystadenoma		
3	Benign mucinous cystadenoma		
4	Benign serous cystadenofibroma		
5	Benign serous cystadenofibroma		
6	Benign serous cystadenofibroma		
7	Benign serous cystadenoma		
8	Benign serous cystadenoma		
9	Borderline mucinous tumour	IA	
10	Borderline mucinous tumour	IA	
11	Borderline mucinous tumour	IA	
12	Borderline mucinous tumour	IC	
13	Borderline serous tumour	IC	
14	Clear cell carcinoma	IB	
15	Mixed serous and endometrioid carcinoma	IIIC	III
16	Mucinous carcinoma	IA	II
17	Mucinous carcinoma	IA	II
18	Serous carcinoma	IA	III
19	Serous carcinoma	IIA	III
20	Serous carcinoma	IIB	II
21	Serous carcinoma	IIIC	II
22	Serous carcinoma	IIIC	III
23	Serous carcinoma	IV	II

### Profiling and statistical analysis of ovarian cyst fluid samples

**Figure 1:**
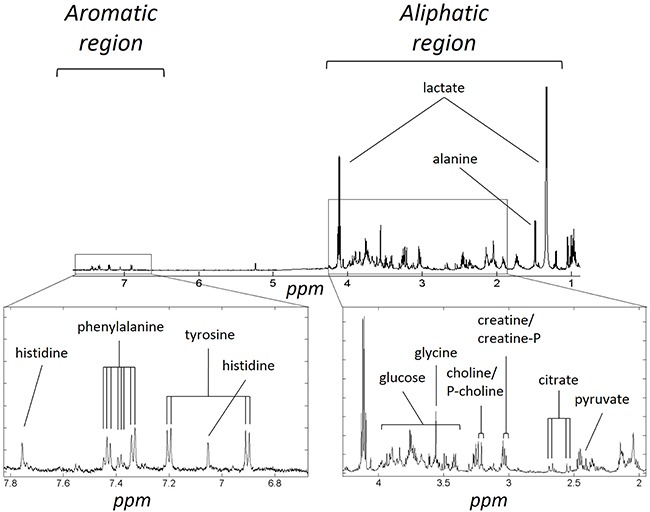
Example of metabolite assignments of a serous carcinoma ^1^H-NMR CPMG metabolic profile The metabolite assignments in the aromatic and aliphatic regions of a representative spectrum are shown above. Peak multiplicity is not indicated.

Figure 1 illustrates an average ovarian cyst fluid CPMG ^1^H-NMR spectrum, belonging to a serous carcinoma sample. An example of assigned identified metabolites including those present in most ovarian cyst fluid metabolic profiles are presented in Table 2. Some metabolites were tumour specific and could be used to distinguish between different types of tumours, such as hypoxanthine which was only detected in a sub cohort of malignant tumours. There were also many unassigned resonances which were consistently observed in the spectra, such as the resonance at ∼2.03 ppm, which is thought to belong to N-acetyl functional groups. This peak was visibly more abundant in the borderline cohort.

**Table 2:** List of assigned metabolites and their respective resonances

Molecule	^1^H-shift (ppm)	Multiplicity	Functional group
acetate	1.92	s	CH_3_
alanine	1.48	d	CH_3_
	3.78	q	CH_2_
choline	3.20	s	N(CH_3_)_3_
citrate	2.56	d	CH_2_
	2.67	d	CH_2_
lysine	1.70	m	CH_2_
	1.88	m	CH_2_
	3.01	t	CH_2_
dimethylamine*	2.72	s	CH_3_
formate	8.46	s	CH
glutamate	2.36	m	γ-CH_2_
α-glucose	3.42	t	H4^†^
	3.54	dd	H2^†^
	5.24	d	H1^†^
glycine	3.56	s	CH_2_
3-hydroxybutyrate	1.20	d	γ-CH_3_
	2.30	m	α-CH_2_
	2.40	m	α-CH_2_
	4.15	m	β-CH
hypoxanthine	8.19	s	H2^†^
	8.20	s	H7^‡^
isoleucine	0.93	t	δ-CH_3_
	1.02	d	β-CH_3_
lactate	1.33	d	CH_3_
	4.11	q	CH_2_
leucine	0.95	d	δ-CH_3_
	0.97	d	δ-CH_3_
	1.72	m	CH_2_
	1.73	m	CH_2_
phenylalanine	7.33	m	H2^†^, H6^†^
	7.38	m	H4^†^
	7.43	m	H3^†^, H5^†^
pyruvate	2.41	s	CH_3_
phosphocholine*	3.21	s	N(CH_3_)_3_
tyrosine	6.91	d	H3^†^, H5^†^
	7.19	d	H2^†^, H6^†^
valine	1.00	d	CH_3_
	1.04	d	CH_3_

† and ‡ denote that the protons are part of a six or five membered ring respectively. s – singlet; d – doublet; dd – double doublet; t – triplet; q – quadruplet; m – multiplet.

* tentative assignment.

**Figure 2:**
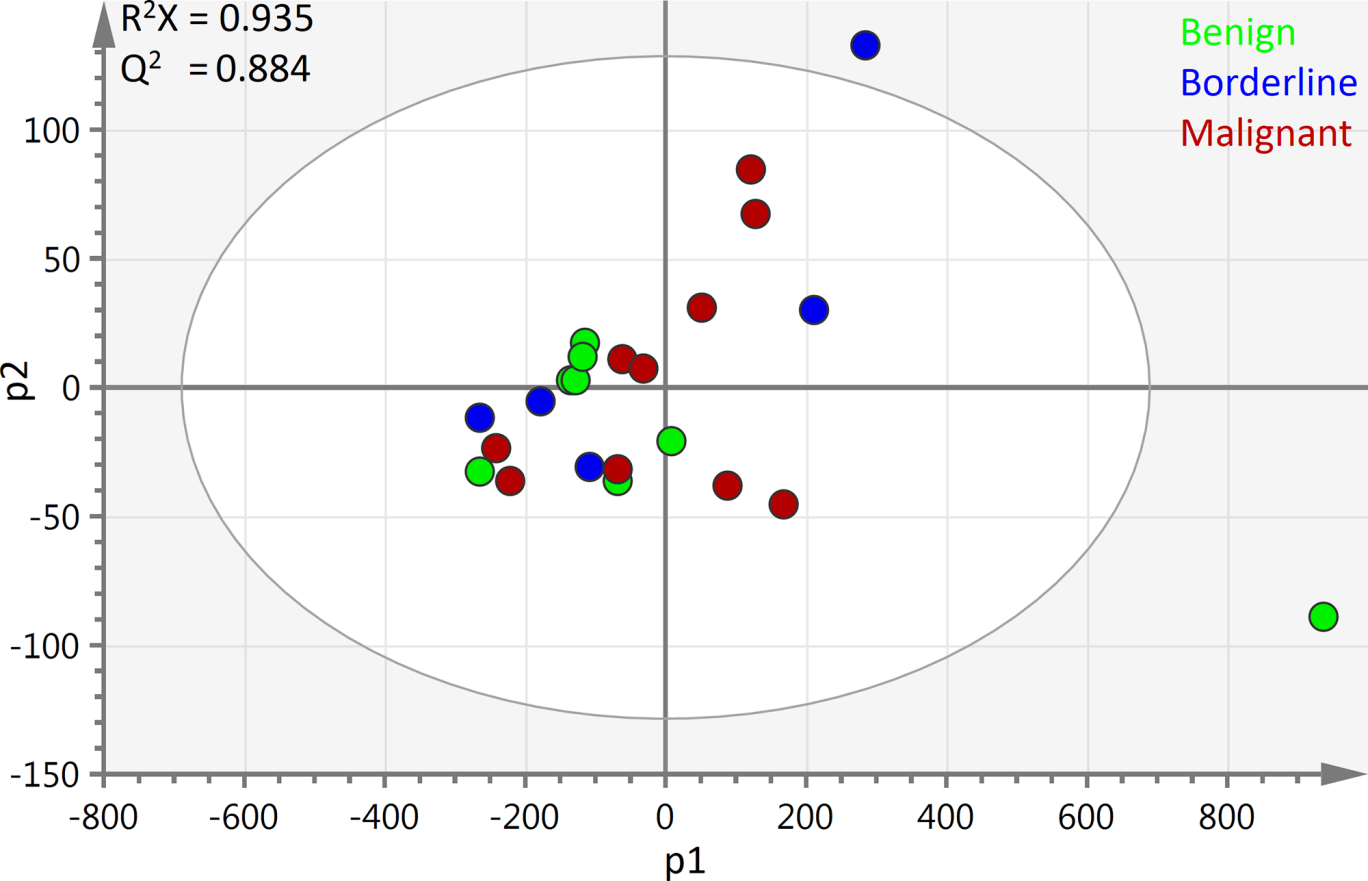
PCA scores plot of all ovarian cyst fluid ^1^H-NMR spectra: The first two principal components of the scores plot of all 23 samples are depicted above The benign samples are shown in blue, borderline are green and malignant are red. R^2^X1 = 0.904 R^2^X2 = 0.0314. Bo – borderline; M – Malignant; Be – Benign; PC1 – first principal component; PC2 – second principal component scores.

PCA showed that the three tumour types could not be separated based on the raw spectral data alone but there was a degree of clustering observed between benign and malignant samples (Figure 2). A waterfall plot of the first principal component scores is illustrated in Supplementary Figure S1.

The loading scores revealed that lactate was at relatively very high levels in a benign mucinous cystadenoma and a borderline mucinous tumour sample but remodelling the ^1^H-NMR data without these two samples did not lead to a significant difference in the statistical parameters or the observed pattern of group clustering, while lactate remained a strong discriminatory feature. Modelling the data with supervised multivariate analyses was also attempted but the resulting models were of low predictive ability and were not statistically significant.

**Figure 3:**
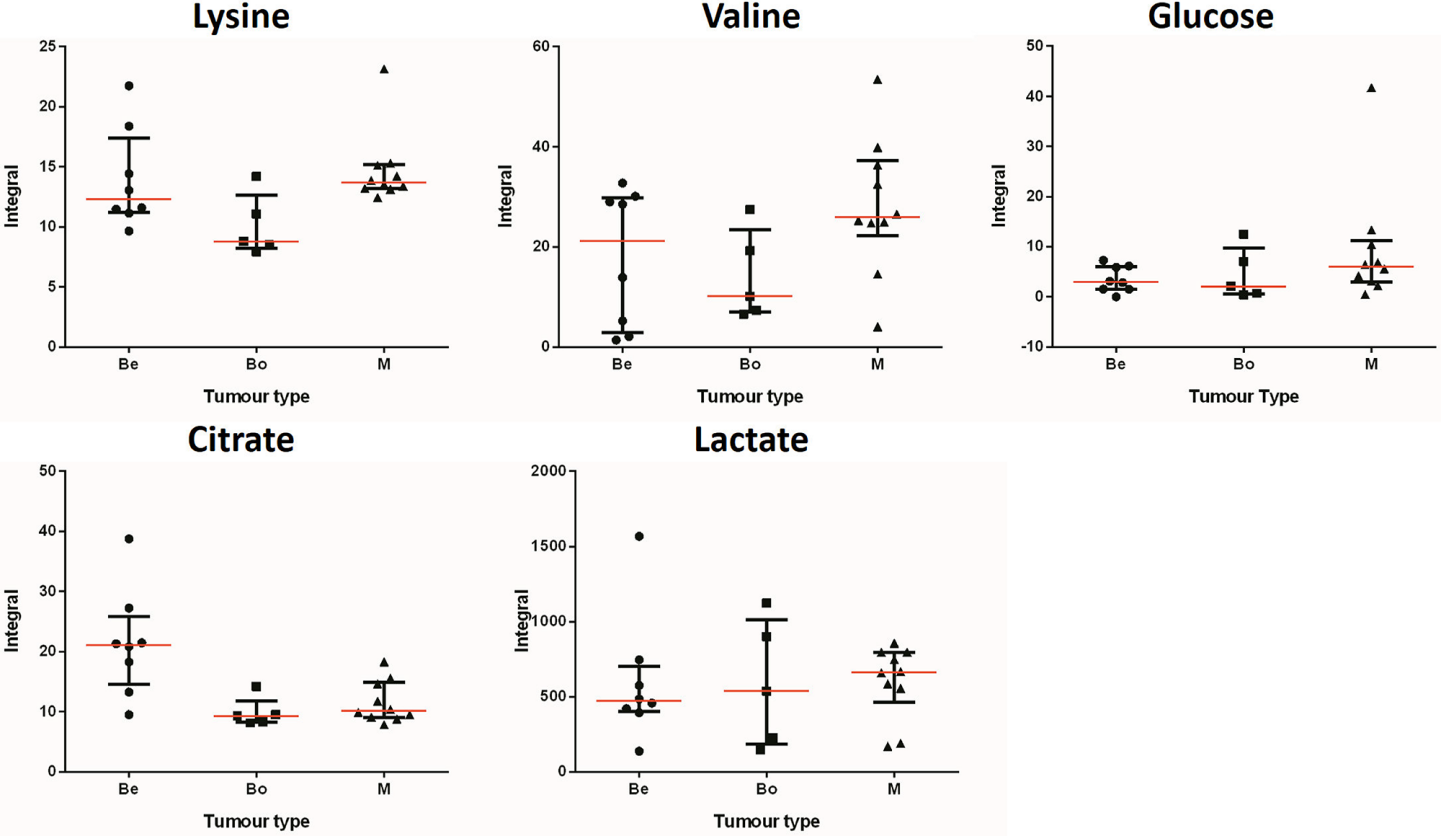
Integral values, medians and median range of key metabolites The integral values from all 23 samples for each metabolite are shown above. The samples are separated by tumour type: benign (Be), borderline (Bo) and malignant (M). Each point on the graphs represents an integral value. The median range is indicated by the error bar, while the median value for each metabolite and group is indicated by the red horizontal line in the middle of the error bar.

Univariate analysis was used to compare the integral levels of acetate, alanine, choline, citrate, lysine, 3-hydroxybutyrate, glucose, leucine, phenylalanine, hypoxanthine and valine. The calculated median and interquartile range of each metabolite in each tumour group are presented in Table 3, while Figure 3 illustrates the integral, median and interquartile range values of citrate, lysine, lactate, glucose and valine in each sample in each group. Supplementary Table S1 presents the integrals of all analysed metabolites. The univariate analysis revealed that the higher levels of citrate in the benign samples were statistically significant with a p-value of 0.0085. They also revealed that the higher levels of lysine in the malignant samples were statistically significant when compared to the benign and borderline group, with a p-value of 0.0439.

**Table 3:** Median values and interquartile range of each metabolite integral in each cohort

Metabolites	Benign	Borderline	Malignant
Acetate	8.53 (6.35)	5.35 (1.12)	9.56 (3.66)
Alanine	43.52 (49.93)	22.67 (24.70)	53.09 (31.33)
Choline	10.56 (1.99)	9.48 (7.13)	13.87 (31.33)
**Citrate**	**21.08 (5.89)**	**9.27 (1.22)**	**10.15 (4.73)**^†^
**Lysine**	**12.34 (4.03)**	**8.80 (2.56)**	**13.72 (1.66)**^‡^
3-Hydroxybutyrate	18.14 (7.39)	20.92 (7.02)	16.29 (5.83)
Glucose	2.98 (4.42)	2.086 (6.36)	6.02 (6.10)
Hypoxanthine	n.d	n.d	2.61 (0.85)
Lactate	471.81 (203.11)	538.05 (671.48)	663.67 (219.39)
Leucine	41.70 (39.55)	24.09 (10.46)	51.48 (25.80)
Phenylalanine	4.01 (5.72)	1.97 (1.85)	4.73 (5.70)
Valine	21.22 (24.81)	10.16 (11.96)	25.91 (10.61)

The median values and interquartile range (in parentheses) of the integrals of selected metabolites from each cohort are shown.

† and ‡ indicate stat. significance between benign vs. malignant and benign/borderline vs. malignant respectively.

Hypoxanthine was detected in only two malignant samples.

## DISCUSSION

In the current work we sought to gain further insight into the metabolic profile of the ovarian tumour micro-environment. The diversity of protein content is one of the most important reasons why ovarian cyst fluid samples should be a rich medium for metabonomic analysis. It is not possible to ascertain whether the metabolic profile of the cyst fluid is identical to that of the tumour cells or whether it has a separate metabolic signature. However, different tumour types are likely to have specific protein, lipid and metabolite content which will obviously influence their metabolic profile and help distinguish between them both via analysis of tumour cells or tumour cyst fluid.

In addition to the identified assigned metabolites present most ovarian cyst fluid metabolic profiles, there were many unassigned resonances which were consistently observed in the spectra. One example is the resonance at ∼2.03 ppm, which was also unassigned by *Boss et al.* and is thought to belong to N-acetyl functional groups. [10] Even though this peak was visibly more abundant in the borderline cohort, it was not further analysed quantitatively since it was not possible to integrate the peak accurately due to its broadness. *Kolwijck et al.* originally had assigned N-acetylaspartate in that region in non-borderline tumours, [20] as also reported and further investigated in a more recent publication, [21] but we could not detect the remaining resonances of that compound and confirm the assignment. However, it was also suggested that other N-acetyl functional groups from glycosylated proteins or lipids have resonances in this region, which could account for the observed resonance and future studies with greater borderline tumour sample numbers could lead to the identification of a borderline tumour specific macromolecule. [20]

The borderline tumour group was not observed to have any visible group specific metabolic patterns, apart from the N-acetyl resonance. However, some resonances, including those of the amino acids were at lower levels when compared to the benign group but this could not be explained. In the case of the malignant group, the qualitative analysis revealed that hypoxanthine was only identified in two malignant samples but not in any other tumour type. Hypoxanthine is a purine derivative whose nucleoside form is inosine. It has already been proposed as a urine biomarker for non-Hodgkin's lymphoma, [22] while it has also been reported to exist in higher levels in plasma and to have reduced excretion in gastric and colorectal tumours. [23]

Our results show the presence of lysine was at higher levels in the malignant tumours when compared to the non-malignant tumours, while citrate was depleted in the malignant tumours when compared to benign tumours. Higher levels of lysine in malignant ovarian tumours have been previously reported by *Boss et al.* but the biochemical mechanism leading to this increase is not known. [10] Citrate is formed in the mitochondria and is involved in the Krebs cycle, which is an integral part of aerobic respiration. Cytosolic citrate is also used to form acetyl-CoA by the enzyme ATP-citrate lyase which is subsequently used in fatty acid synthesis. Fatty acid synthesis is an essential process in many tumour cells to permit rapid growth and its inhibition has been shown to delay tumour progression in a xenograft model of ovarian cancer, amongst others. [24] Furthermore, the inhibition of ATP-citrate lyase, an important enzyme in fatty acid biosynthesis, has also been previously reported to suppress tumour growth. [25] Therefore, the importance of citrate in fatty acid synthesis might be linked to its observed depletion in the malignant tumours.

While no statistically significant difference was observed between the levels of acetate, alanine, valine, phenylalanine, leucine, glucose, choline, lactate and 3-hydroxybutyrate between tumour groups, the integral clustering and medians of valine, leucine, alanine, glucose, choline and lactate were generally higher in the malignant samples and could be potentially distinguishing. The absence of statistical significant difference in the levels of these metabolites could be due to the significant heterogeneity in the tumour types and FIGO stages, coupled to the small sample numbers per tumour group. The observed intragroup heterogeneity for citrate, lysine, glucose, lactate and valine is in agreement with what was previously reported. [10]

Lactate was one of the metabolites that were initially expected to exist at higher levels in malignant samples due to the Warburg effect. [17] Higher levels of lactate dehydrogenase in the peritoneal fluid have been suggested to be a prognostic biomarker for epithelial ovarian cancer. [26] An increase in the levels of glucose could also be explained since a rise in the expression of glucose transporter 1 (GLUT1), a transporter responsible for glucose uptake, has been reported in ovarian carcinomas. [27] Finally, an increased uptake of choline and synthesis of phosphocholine has also been observed and it was a distinguishing factor between epithelial ovarian cancer cells and immortalized epithelial ovarian cells. [28] This has recently been proved to be of utility in patient imaging using contrast-enhanced magnetic resonance imaging and 3D chemical shift imaging with strong potential for use in clinical practice for diagnosis of ovarian cancer. [29]

**Figure 4:**
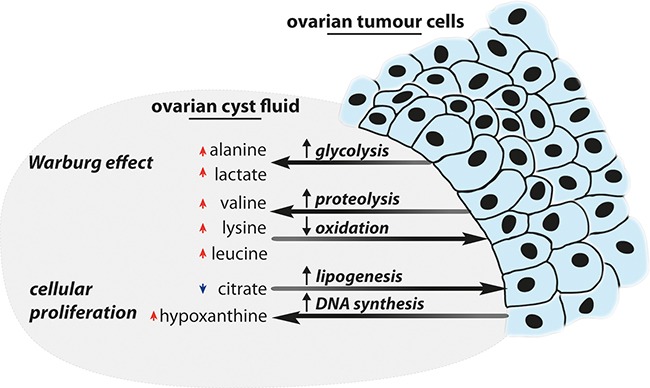
Summary of interpreted metabolic perturbations An overview of the metabolic perturbations identified in the ovarian cyst fluid, and how they are linked to ovarian tumours, is displayed above. Please note that red arrows indicate elevated levels for a specific metabolite and vice versa for blue metabolites.

The observed higher levels of lysine, 3-hydroxybutyrate, valine, lactate and choline in the malignant tumours are in agreement with previous metabolic profiling studies of ovarian cyst fluid samples. [10, 21] However, there are also differences reported in the literature that we did not observe, including higher levels of glutamine, methionine and threonine in the malignant tumour group. [10] Furthermore, a previous investigation also reported a depletion of glucose, which would be in line with the Warburg effect and is in contrast to what we have observed. [17] This difference between the two studies should be investigated further and could simply be due to the reported great variability in the metabolite concentration that exists in the cyst fluid samples, that was observed in our investigation as well. [10] A summary of the main metabolic perturbations is illustrated in Figure 4.

This is the first metabonomic study to include benign, malignant as well as borderline ovarian tumours in a high throughput broad ^1^H-NMR spectroscopic analysis of ovarian tumour cyst fluid. Our analysis supports the conclusion that ovarian cyst fluid is a rich source of information about the metabolic state and nature of the tumour, however, a direct comparison with the metabolic profiles of ovarian tumours would be required to confirm this. In future work it would be valuable to compare profiles from ovarian tumours, ovarian cyst fluid, serum and urine to establish the value of each sample matrix for prognostic and mechanistic information. Several past analyses on plasma/serum by ^1^H-NMR and LC-MS have already demonstrated that metabolic profiling can distinguish between early stage and late stage epithelial ovarian tumours [30], benign and malignant tumours [31], as well as patients with early stage tumours and healthy controls. [32] Furthermore, it has already been shown that the LC-MS metabolic profile of urine can also be used to distinguish between benign and malignant tumours. [33]

An array of metabolites in cyst fluid has been identified with potential to discriminate between benign, borderline and malignant tumours, which merit further investigation and validation with a larger sample cohort. For such a validation study, a targeted metabonomic approach by LC-MS or GC-MS could be employed to investigate the affected metabolic pathways in greater detail in order to obtain more robust mechanistic information. This study overall highlights the potential of the high throughput ^1^H-NMR spectroscopy metabonomic analysis of ovarian cyst fluid in identification of biomarkers that can contribute to the identification and clinical management of all three classes of ovarian tumours.

## MATERIALS AND METHODS

Cyst fluid samples from 23 ovarian epithelial tumours were studied, including 8 benign (Be), 5 borderline (Bo) and 10 malignant (M) tumours.

### Sample procurement, processing and storage

Ethical approval was granted by the Hammersmith and Queen Charlotte's & Chelsea Hospitals Research Ethics Committee. All ovarian cyst fluid samples were collected from resected ovarian tumours within 15 minutes of surgical resection, where the intact cysts were immediately transferred from the operative theatre to the histopathology laboratory at the Hammersmith Hospital in London. Cyst fluid was drained from the cyst using a syringe or via a small puncture in the cyst wall and deposited into tubes. On average 100 ml of cyst fluid was drained from each patient. The collected fluid was then centrifuged at 4°C for 10 minutes and the supernatant aliquoted and stored at −80°C. Throughout the process from collection to storage the samples were consistently kept on ice in order to minimize any sample content degradation.

### Metabolite extraction method

#### Methanol extraction

Cold methanol was mixed with the samples at 1:1 ratio and the mixture was incubated at 4°C for 30 minutes, after vortexing. The mixture was then centrifuged for 10 minutes at 12000 g (Pico microcentrifuge, Thermoscientific, Waltham, MA, USA). The resulting supernatant was dried overnight in a centrifugal concentrator (SpeedVac, Thermoscientific).

### ^1^H-NMR metabolic profiling of ovarian cyst fluid

The dried supernatants from all metabolite extraction method were then re-dissolved in 600 μl of 0.2 M phosphate buffer solution. Following resuspension, the mixture was centrifuged for 5 minutes at 12000 g and 550 μl was transferred to a 5 mm NMR tube for analysis (Norell NMR Precision tube 507-HP-7; Norell Inc, Marion, NC, USA). For the metabonomic analysis of single pulse ^1^H-NMR experiments were performed using a Bruker DRX600 spectrometer (Bruker) operating at 600 MHz (14.1 T) at a temperature of 300 K, with a broadband inverse probe and an automated delivery system. The D_2_O present in the buffer provided the field frequency lock while the TSP was used as a chemical shift reference. 64 and 256 scans were performed for the standard one dimensional water pre-saturation (NOESYpr1d) and Carr-Purcell-Meiboom-Gill (CPMG; 2nτ of 64 ms (*n* = 160, τ = 200 μs)) experiments, respectively, and 32697 data points were recorded for all experiments at a spectral width of 12000 Hz. The Carr-Purcell-Meiboom-Gill (CMPG) pulse sequence [34] allows transverse (T_2_) relaxation of nuclear polarization to occur while refocusing the evolution of chemical shift and other sources of inhomogenous broadening of spins. Since larger molecules have shorter T_2_ times, allowing a degree of T_2_ relaxation before signal acquisition effectively reduces the contribution of high-molecular weight species (such as protein) to the final NMR spectrum relative to that of small molecules (such as metabolites). [15]

For spectrum evaluation and Fourier transformation of the free induction decay (FID) the TopSpin software (Bruker) was used. The assignment of hypoxanthine was confirmed by spike-in experiments with the pure compound.

### Data processing and statistical analysis

Assignment of metabolites from the ^1^H-NMR metabolic profiles was performed using Chenomx NMR suite (Chenomx, Edmonton, Canada) and the human metabolome database (www.hmdb.ca). For processing, the TopSpin software (Bruker) was used for initial spectral processing followed by the software package of MATLAB (Mathworks, Natick, MA, USA). Probabilistic quotient normalization was employed prior to statistical analysis and calculation of integrals. [35]

Principal component analysis (PCA) on SIMCA-P+ 12.01 (Umetrics, Umea, Sweeden) was used as a multivariate statistical approach to observe how the relationship of the samples in multivariate space. The ^1^H-NMR spectra were divided into three groups, depending on the tumour type of the sample. Mean centring with no scaling was chosen and PCA was performed on the first two principle components. Mann-Whitney tests were used to analyse the different levels of twelve metabolites between the three groups at a 5% significance level (Prism; GraphPad, La Jolla, CA, USA). The group comparisons on which the Mann-Whitney tests were used were: benign versus malignant tumours and benign and borderline versus malignant tumours.

## SUPPLEMENTARY FIGURE AND TABLES


