# Supplementary material for: Metabonomic analysis of ovarian tumour cyst fluid by proton nuclear magnetic resonance spectroscopy
Source: Oncotarget. 2016 Jan 12;7(6):7216–26. doi: 10.18632/oncotarget.6891 (PMC4872780; doi:10.18632/oncotarget.6891)
Supplement: Supplementary file 1 [file oncotarget-07-7216-s001.pdf]

SUPPLEMENTARY FIGURE AND TABLES

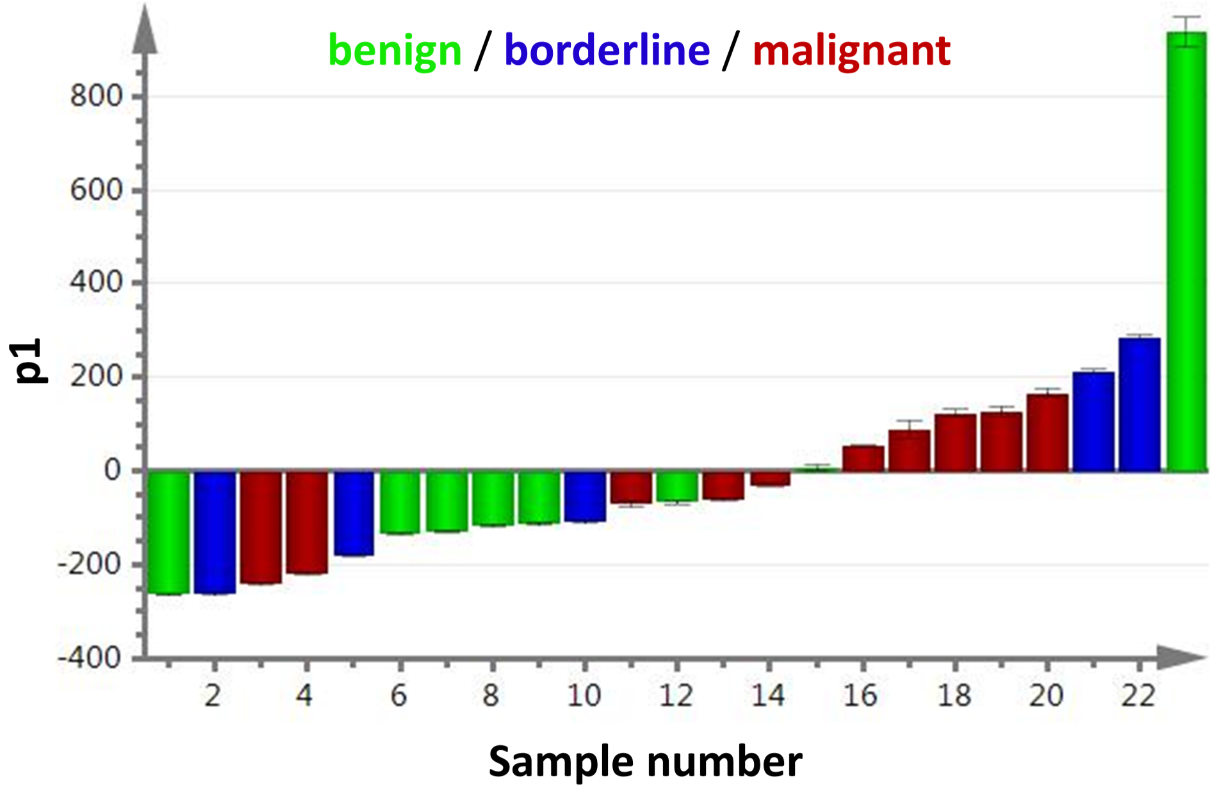

Supplementary Figure S1: Waterfall plot of first principal component. A waterfall plot displaying the first principal component scores of the principal component analysis consisting of all samples. The scores have been ordered in an ascending order. Benign samples are coloured green, borderline samples are coloured blue and malignant samples are coloured red.

Supplementary Table S1: Integral values of all analysed metabolites from ^{1}H-NMR ovarian cyst fluid metabolic profiles

Tumour group	Sample number	Metabolites						
		acetate	alanine	choline	citrate	glucose	3-HB	hypoxanthine
Benign	1	8.07	14.82	8.24	20.84	6.14	20.62	0
	2	20.52	31.34	8.29	13.29	7.28	17.58	0
	3	15.87	55.69	10.46	9.51	5.91	18.70	0
	4	11.24	15.59	51.07	27.25	0.00	28.45	0
	5	5.39	73.44	11.88	21.32	1.52	14.14	0
	6	9.00	65.43	11.01	18.28	3.11	12.14	0
	7	6.26	65.29	10.66	21.47	1.56	12.41	0
	8	3.85	6.33	9.56	38.77	2.84	22.53	0
Median		8.53	43.52	10.56	21.08	2.98	18.14	0
IQR (1st)		6.04	15.40	9.24	17.03	1.55	13.70	0
IQR (3rd)		12.40	65.33	11.23	22.92	5.97	21.10	0
Borderline	9	6.11	40.89	6.01	8.16	7.05	15.50	0
	10	4.97	22.67	7.63	9.27	0.43	22.52	0
	11	4.99	9.11	14.76	8.29	2.09	33.05	0
	12	5.35	16.19	18.09	14.19	12.52	20.92	0
	13	8.21	56.83	9.48	9.51	0.70	12.06	0
Median		5.35	22.67	9.48	9.27	2.09	20.92	0
IQR (1st)		4.99	16.19	7.63	8.29	0.70	15.50	0
IQR (3rd)		6.11	40.89	14.76	9.51	7.05	22.52	0
Malignant	14	11.54	62.55	7.313346	10.40	4.18	12.66	
	15	6.58	30.52	8.740056	7.83	41.77	147.95	0
	16	6.37	29.19	8.725457	14.67	6.44	16.51	0
	17	13.47	39.03	8.70755	9.11	10.42	20.39	0
	18	7.96	64.46	13.63562	9.90	0.47	16.07	0
	19	10.25	74.24	16.37562	9.51	3.16	14.97	1.76
	20	10.70	93.09	14.11108	15.60	2.25	6.45	0
	21	9.15	46.87	18.59697	8.82	13.32	15.52	3.45
	22	4.24	8.33	15.24608	18.32	5.61	34.83	0
	23	9.97	59.31	14.28195	11.74	6.80	21.12	0
Median		9.56	53.09	13.97	10.15	6.03	16.29	0
IQR (1st)		6.93	32.65	8.73	9.21	3.42	15.11	0
IQR (3rd)		10.59	63.98	15.01	13.94	9.52	20.94	0

(*Continued*)

Tumour group	Sample order	Metabolites				
		lactate	leucine	lysine	phenylalanine	valine
Benign	1	393.50	19.67	18.39	1.38	5.25
	2	421.19	23.22	11.48	2.22	13.89
	3	745.26	47.89	14.44	6.76	28.99
	4	138.21	35.49	21.75	0.00	2.13
	5	485.42	60.37	11.15	5.81	28.56
	6	458.19	66.41	13.06	6.95	32.77
	7	574.76	69.55	9.65	6.92	30.15
	8	1567.00	10.21	11.62	0.17	1.40
Median		471.80	41.09	12.34	4.01	21.22
IQR (1st)		414.27	22.33	11.40	1.08	4.47
IQR (3rd)		617.38	61.88	15.43	6.80	29.28
Borderline	9	899.94	30.44	11.08	3.73	19.28
	10	1123.19	14.94	14.22	1.97	6.58
	11	228.47	19.97	7.95	1.07	7.33
	12	145.28	24.09	8.52	1.88	10.15
	13	538.05	66.03	8.80	6.64	27.48
Median		538.05	24.09	8.80	1.97	10.15
IQR (1st)		228.47	19.97	8.52	1.88	7.33
IQR (3rd)		899.94	30.44	11.08	3.73	19.28
Malignant	14	796.12	65.21	13.21	9.67	32.57
	15	170.94	42.14	13.13	2.81	24.77
	16	586.75	32.87	13.56	3.24	14.62
	17	659.48	40.24	13.88	3.82	26.54
	18	795.24	52.28	13.38	4.39	24.99
	19	747.87	69.72	12.46	9.60	39.87
	20	667.86	98.47	23.17	14.68	53.51
	21	556.43	50.69	15.32	5.08	25.28
	22	190.74	20.01	15.14	0.49	4.05
	23	857.24	66.94	14.24	7.53	36.38
Median		663.67	51.49	13.72	4.74	25.91
IQR (1st)		564.01	40.72	13.25	3.39	24.83
IQR (3rd)		783.40	66.51	14.92	9.08	35.43
